# FGF negative regulation during early myogenesis

**DOI:** 10.1186/1471-2164-15-S2-O23

**Published:** 2014-04-02

**Authors:** Muhammad Abu-Elmagd, Andrea Münsterberg

**Affiliations:** 1 Center of Excellence in Genomic Medicine Research (CEGMR), King Abdulaziz University, P.O. Box: 80216, Jeddah 21589, Kingdom of Saudi Arabia; 2 Minia University, Faculty of Science, Zoology Department, El-Minia, 61519, Egypt; 3 University of East Anglia, School of Biological Sciences, Department of Cell and Developmental Biology, Norwich, NR4 7TJ, UK

## 

Negative regulators of signal transduction cascades play critical roles in controlling different aspects of normal embryonic development. Sprouty2 negatively regulates FGF signaling and Receptor Tyrosine Kinases (RTK) and is important in differentiation, cell migration and proliferation. In vertebrate embryos, Sprouty2 is expressed in pre-segmented mesoderm and in forming somites. Expression is maintained in the myotome until late stages of somite differentiation. However, its role and mode of action during somite myogenesis is still unclear. In the current study, we analysed chick Sprouty2 expression and showed that it overlaps with that of Myogenic Regulatory Factors (MRF) MyoD and Mgn. Targeted mis-expression of Sprouty2 led to inhibition of myogenesis, whilst its C-terminal domain interference led to an increased number of myogenic cells by stimulating cell proliferation. Our results show that Sprouty2 plays crucial role in regulating chick myogenesis by fine tuning of FGF signaling through a negative feedback loop.

